# Nonoperative treatment of Maisonneuve fractures

**DOI:** 10.1007/s00068-026-03229-x

**Published:** 2026-06-08

**Authors:** Jan Frühauf, Jan Bartoníček, Petr Fojtík, Anna Horňáková, Michal Tuček

**Affiliations:** 1 Department of Orthopaedics, First Faculty of Medicine, Charles University and Military University Hospital Prague, U Vojenské Nemocnice 1200, Prague 6, 169 02 Czech Republic; 2 Institute of Hygiene and Epidemiology of the 1st Faculty of Medicine and General, University Hospital in Prague, Studničkova 7, Prague 6, 128 00 Czech Republic

**Keywords:** Maisonneuve fracture, Medial malleolus, Posterior malleolus, Fibular notch, Tibiofibular syndesmosis

## Abstract

**Purpose:**

The aim of this study is to describe the basic pathoanatomical characteristics of a stable Maisonneuve fracture and mid-term results of its nonoperative treatment.

**Methods:**

The study included 17 prospectively collected patients with a mean age of 59 years. The postinjury ankle CT had to meet the following criteria: nondisplaced or minimally displaced (up to 1 mm) fracture of medial malleolus, medial clear space less than 3 mm, nondisplaced or minimally displaced (up to 2 mm) fracture of posterior malleolus, anatomical position or minimal malposition of the distal fibula in the fibular notch (widening of the tibiofibular space up to 2 mm or external rotation of the distal fibula up to 10°). The average follow-up period was 34 months, the final follow-up included CT examination and functional evaluation based on AOFAS and FAOS scores.

**Results:**

A medial malleolus fracture was recorded in 12% cases, a posterior malleolus fracture in 29% patients and a Tillaux-Chaput tubercle fracture in 18% cases. All fractures of the proximal fibula, medial and posterior malleolus healed within 3 months. The position of the distal fibula in the fibular notch did not change in 11 cases compared to the post-injury CT scan, improved slightly in 5 cases, and worsened slightly in 1 case. The average final AOFAS hindfoot score was 96.9 points and the average final FAOS score was 98.7%.

**Conclusion:**

A stable form of the Maisonneuve fracture is characterized by no or minimal displacement on CT scans and can be successfully treated nonoperatively.

## Introduction

Maisonneuve fracture (MF), an ankle fracture-dislocation associated with a fracture of the proximal quarter of the fibula [1], was characterized by Bonin [2] as a stable injury. On the contrary, B.G. Weber [3] considered MF to be an unstable fracture combined with the rupture of all ligaments of the tibiofibular syndesmosis and the interosseous membrane up to the level of the fracture on the fibula. This was supposed to lead to both instability of the tibiofibular mortise and proximal displacement of the fibula relative to the distal tibia and the talus with disruption of the so-called Weber nose and Weber circle [4]. According to AO/ASIF, these injuries were clearly indicated for surgical treatment [3]. Nevertheless, some American authors treated even these fractures nonoperatively [5, 6].

Recent studies based on CT and MRI examinations have considerably changed the view of MF pathoanatomy [7–14]. Among other things, these studies have demonstrated that there are stable MFs whose pathoanatomy does not correspond to the Weber’s concept [3]. However, only a few case reports confirming very good results of nonoperative treatment of MF have been published in the literature [15–19]. An evaluation of a larger patient cohort is still lacking.

## Materials and methods

The study was done in accordance with the Declaration of Helsinki, approved by Ethics Committee of the Military University Hospital Prague (reference number: 108/16–35/2021) and patients consented to participate in the study.

### Material

Between January 1, 2014, and December 31, 2023, we treated at our Department 182 patients with a Maisonneuve fracture (MF), who were then prospectively followed up [9, 10, 12, 14]. The criteria for inclusion of patients in the study included an ankle fracture-dislocation associated with a fracture in the proximal quarter of the fibula and combined with additional injuries in the ankle region. Specifically, these injuries involved ligaments of the tibiofibular mortise, medial structures (MS), i.e., the deltoid ligament (DL) and the medial malleolus (MM), and the posterior malleolus (PM). Exclusion criteria included immature skeleton, previous fractures in the ankle region, osteoarthritis of the ankle at the time of injury and incomplete radiographic documentation.

Out of 182 patients with MF, we identified a total of 17 patients (8 men and 9 women) who were indicated for nonoperative therapy after radiographic and CT examinations. The mean age of the whole cohort was 59 years (range; 30–84 years), with men averaging 49 years (range; 30–76 years) and women 68 years (range; 47–84 years). The right side was affected in 7 cases, the left side in 10 cases. The average follow-up period was 34 months (range;12–122 months) (Table 1).

**Table 1:** Patient cohort – basic information

tients *N*	Age (y)	G	Side	Fx MM	Fx PM	Fx T-Ch	FN Inj	FN FU	FU (M)	AOFAS	FAOS (%)
1 *	30	M	L	-	-	-	W	NC	17	100	100
2	38	M	R	-	-	-	ER	NC	122	93	96
3	39	M	R	-	3	-	W	D	31	100	99
4	41	M	R	-	-	-	A	NC	26	100	100
5 *	43	M	R	-	-	-	W	I	20	100	100
6	47	F	R	-	4	-	A	NC	94	100	100
7	54	F	L	-	-	-	A	NC	17	100	100
8 *	57	M	L	-	-	-	W	I	14	87	100
9 *	64	F	L	-	-	1	W	NC	14	100	100
10 *	65	F	R	-	2	1	W	NC	24	100	100
11	65	M	R	-	-	-	ER	NC	59	100	100
12	70	F	L	BC	2	-	ER	I	12	100	100
13	73	F	L	-	3	-	ER	I	40	85	93
14	74	F	L	-	-	1	ER	I	16	95	98
15	76	M	L	BC	-	-	A	NC	47	100	100
16	78	F	R	-	-	-	A	NC	12	97	98
17 *	84	F	L	-	-	-	ER	NC	19	90	94

* Patients examined by MRI, M - male, F - female, L - left, R - right, Fx - fracture, MM - medial malleolus, BC - bicollicular, PM - posterior malleolus, Ti-Ch - Tillaux-Chaput tubercle, FN Inj - position of distal fibula in FN after injury, FN FU - position of the distal fibula at the last CT follow-up, A – anatomical position, W- widening, ER - external rotation, NC - not changed, I - improved, D - deteriorated, FU – follow up, M – months

### Methods

Radiographs of the ankle were obtained in all patients in three (anteroposterior, lateral and mortise) views, and radiographs of the lower leg in two (anteroposterior and lateral) views. After application of a below-knee cast with the ankle in the neutral position, CT scans according to our standard protocol (in the axial, frontal and sagittal planes) and 3D CT reconstructions were performed in all the patients. A total of 6 patients underwent MRI examination of the ankle (Table 1).

**Fig. 1:**
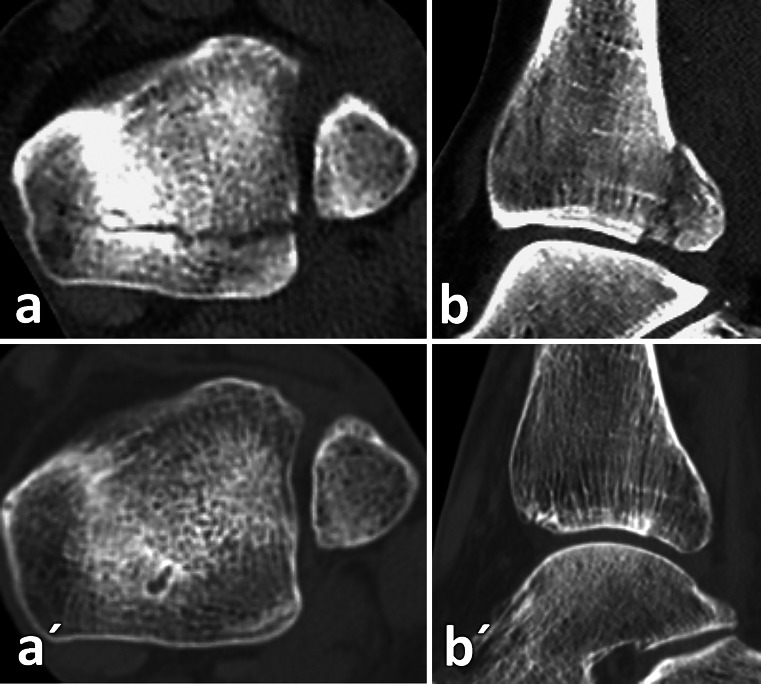
Patient N-13. **a** – an axial postinjury CT scan fracture shows 10 degrees of external rotation of the distal fibula in FN and fracture of PM type 3 according B-R classification; **b** – a sagittal postinjury CT scan with 2 mm proximal displacement of PM; **a´** - an axial follow-up CT scan 12 months after injury, external rotation of the distal fibula in FN is smaller; **b´**- a sagittal follow-up CT scan 12 months after injury fracture shows healing of PM in an almost anatomical position

Nonoperative treatment was indicated for patients who met the following criteria:

- Nondisplaced or minimally displaced (up to 1 mm) fracture of MM, and / or medial clear space (MCS) on frontal and axial CT scans of less than 3 mm,
- Nondisplaced or minimally displaced (up to 2 mm) fracture of PM (Fig. 1) on axial CT scans at the level of ankle joint line,
- Anatomical position or minimal malposition of the distal fibula in the fibular notch (FN), i.e. widening of the tibiofibular space up to 2 mm and / or external rotation of the distal fibula up to 10 degrees.

The treatment consisted in applying a below-knee plaster cast for 6 weeks. Radiographic follow-ups were performed at 2 weeks, 6 weeks, 3 months, 6 months, and 12 months after the injury. At the final follow-up, which took place in 2025, a radiograph and CT scan were obtained and the functional results of the treatment evaluated.

### Evaluation

Evaluation focused on fracture pathonanatomy, i.e., the type of fracture of the proximal fibula, injuries to the medial structures (MM and DL), anatomy of the lateral gutter (relationship between the distal fibula and the talus dome), PM fracture type according to the Bartoníček-Rammelt (B-R) classification [20], type of fracture of the Tillaux-Chaput (T-Ch) tubercle according to the Rammelt classification [21] and position of the distal fibula in the fibular notch (FN) according to Bartoníček et al. [9 Bar]. The evaluation of radiological results included healing time, the relationship between articulating bones, especially the position of the distal fibula in FN on axial CT scans.

Functional results were evaluated according to the AOFAS and FAOS scores [22, 23].

Pathoanatomical and radiological results were evaluated individually by each author in the first round and collectively by all authors in the second round. Functional results were evaluated by the first author of the study.

### Statistical analysis

Statistical analysis was performed using the **R** program (version 4.4.2). *Nonparametric* tests were used to compare AOFAS scores between individual categories, i.e., the Mann-Whitney U test for comparing two groups and the Kruskal-Wallis test for comparing more than two groups. The level of statistical significance was set at *p* < 0.05. Furthermore, a correlation analysis between the AOFAS and FAOS scores was performed using the Spearman’s correlation coefficient.

## Results

### Fracture pathoanatomy

Fibula fractures were always subcapital, simple nondisplaced or minimally displaced (up to 2 mm) spiral fractures. In 4 cases, the fracture was not visible in the anteroposterior view.

**Fig. 2:**
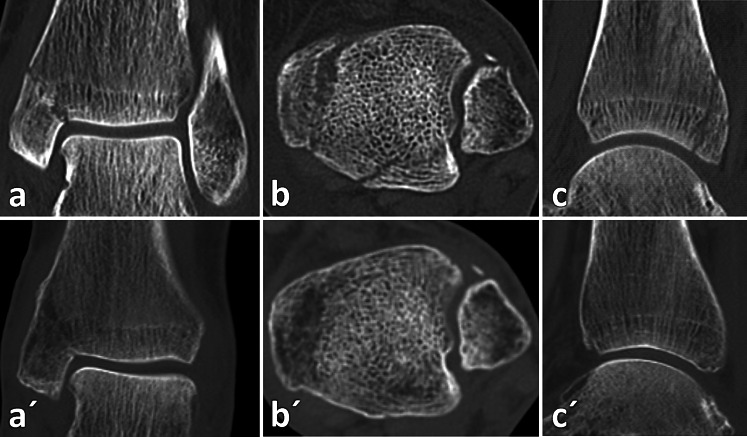
Patient N-12. **a; b; c** – frontal, axial and sagittal postinjury CT scans demonstrate a nondisplaced bicollicular fracture of MM, a nondisplaced fracture of PM type 2 according to B-R classification and 10 degree external rotation of the distal fibula in FN; **a; b; c** – frontal, axial and sagittal CT scans 40 months after injury, all fractures healed in an anatomical position, external rotation of distal fibula improved

Injuries to the medial structures (MS) was recorded only in 2 (12%) cases, i.e., a nondisplaced bicollicular fracture of the medial malleolus (Fig. 2). In all 17 cases, MCS was less than 3 mm. In all 6 patients examined by MRI, DL was intact (Table 1).

**Fig. 3:**
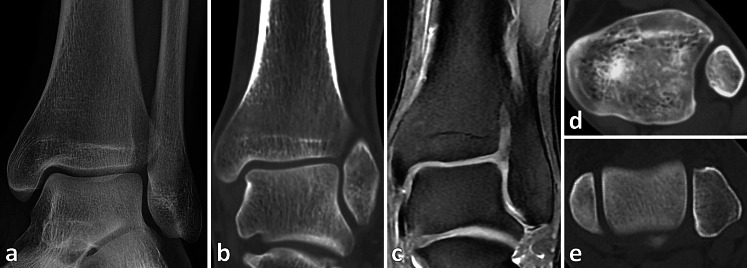
Patient N- 5. **a** – a mortise view; **b** – a frontal CT scan; **c** – a frontal MRI scan; **d** – an axial CT scan taken 5 mm proximal to the joint line; **e** - an axial CT scan taken 5 mm distal to joint line. Figures a, b, c and e show normal anatomy of the lateral and medial gutters, Figure c shows an intact interosseous tibiofibular ligament, Figure d documents widening of tibiofibular space of 1 mm

Anatomy of the lateral gutter was assessed on the frontal and axial CT scans. In all 17 cases, the relationship between the distal fibula and the talus dome was assessed as anatomical, i.e., the joint space was symmetrical and there were no signs of proximal displacement of the fibula relative to the talus (Fig. 3).

A PM fracture was identified in 5 (29%) patients (Type 2 in 2 cases, Type 3 in 2 cases and Type 4 in 1 case according to the B-R classification).

A fracture of the Tillaux-Chaput tubercle was recorded in 3 (18%) cases, all of which were Type 1 according to the Rammelt classification. In patient N-10, a PM fracture Type 2 of the B-R classification was also found.

**Fig. 4:**
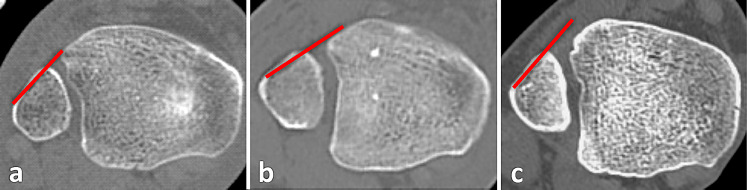
Position of the distal fibula in FN. **a –** normal anatomy, a small triangular space is visible anteriorly; immediately behind it the space narrows and then widens dorsally into a crescent-like area, the anterolateral aspect of the distal fibula still corresponds with the anterior aspect of the distal tibia (red line) ; **b** – widening of the tibiofibular space, the triangular space disappeared but the anterolateral surface of the distal fibula still corresponds with anterior aspect of distal tibia (red line); **c** - external rotation of the distal fibula in FN, the tibiofibular space is anteriorly widened, the anterolateral aspect of the distal fibula does not correspond with the anterior aspect of the distal tibia (red line)

The position of the fibula in FN assessed on an axial CT scan taken 5 mm proximal to the joint line was anatomical in 5 cases, in 6 cases the tibiofibular clear space (TFCS) was widened by 1 to 2 mm, and in 6 cases there was external rotation of the distal fibula by 5–10 degrees, thereby widening the anterior part of the TFCS (Fig. 4).

### Radiological results

All fractures of the proximal fibula, PM, and MM healed within 3 months. During treatment and at the final follow-up, no progression of displacement was observed on radiographs for any of the fractures, and no signs of development of ankle osteoarthritis were recorded.

The final CT scan in all 17 patients showed an MCS value of no more than 2 mm. The anatomy of the lateral gutter was assessed as normal.

**Fig. 5:**
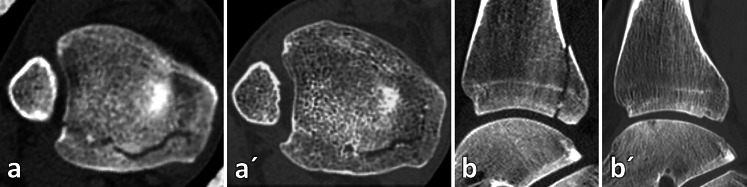
Patient N-3 – widening of the tibiofibular space at the final follow-up. **a** – an axial postinjury CT scan shows widening of the tibiofibular space of 1 mm and a fracture of PM type 3 according B-R classification; **a´** - an axial follow-up CT scan 41 months after injury, documenting widening of the tibiofibular space to 2 mm; **b** – a sagittal postinjury CT scan with 1 mm proximal displacement of PM; **b´** - a sagittal follow-up CT scan 41 months after injury shows healing of PM with less than a 2 mm displacement

The position of the distal fibula in FN on the final CT scan at least 1 year after the injury did not change in 11 cases compared to the post-injury CT scan, improved slightly in 5 cases, and worsened slightly in 1 case (widening by 1 mm) (Table 1), (Fig. 5).

### Functional results

The average AOFAS hindfoot score was 96.9 points (range; 85–100), with 11 (65%) patients achieving the maximum score of 100 points. Points were deducted in 3 cases for mild axial deviation with preserved plantigrade gait (6–15° hindfoot valgus deformity), in 2 cases for mild difficulties during recreational activities, in 2 cases for occasional mild pain, and in 1 case for hindfoot instability (patient N-8, pre-traumatic flatfoot and hyperlaxity) [22].

The average FAOS score was 98.7% (range; 93–100%), with 11 (65%) patients achieving a maximum score of 100%. Points for symptoms were deducted in 1 case for swelling, in 2 cases for a snapping feeling in the joint, in 3 cases for an occasional joint locking feeling during movement, and in 2 cases for pain. Minimal difficulty when walking down stairs was observed in 2 patients, and limitation in sports in 1 patient. Two patients reported a deterioration in their quality of life [23].

A total of 10 patients (59%) achieved full both AOFAS and FAOS scores.

### Statistical analysis

**Fig. 6:**
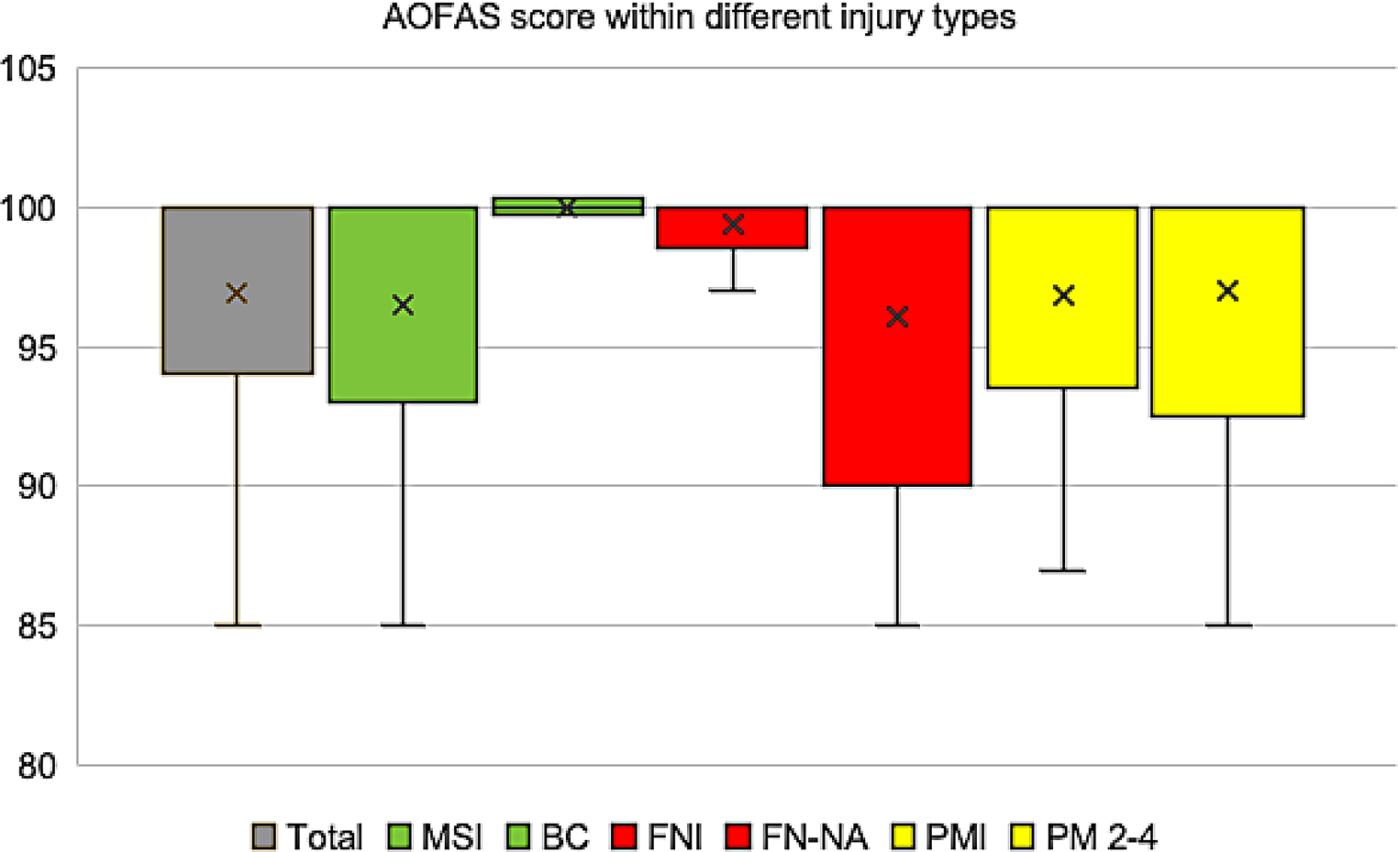
Statistical comparison of functional results of individual injury types. MSI - medial structures intact, BC - bicollicular fracture of medial malleolus, FNI - fibular notch intact, FN-NA - fibular notch non anatomical (widening or external rotation), PMI - posterior malleolus intact, PM 2–4 - posterior malleolus fracture type 2–4 of B-R classification

The AOFAS scores of intact and injured structures of individual fracture pathoanatomy groups were compared (Fig. 6).

- MS (Intact vs. Bicollicular fractures of MM): average AOFAS score 96.5 vs. 100; *p* = 0.578.
- PM (Intact vs. PM fractures): average AOFAS score 96.8 vs. 97.0; *p* = 0.666.
- Position of the distal fibula in FN (Anatomic position vs. Widening or External rotation): average AOFAS score 99.4 vs. 95.8; *p* = 0.141.

The statistical analysis concluded that none of the lesions showed a statistically significant difference compared to intact structures. Nevertheless, Widening a ER groups showed a trend toward a worse functional outcome.

## Discussion

Pankovich [5], in 1976, treated 6 out of 17 MF cases nonoperatively. In all 6 cases the medial structures (DL, MM) were intact; 4 MFs with a PM fracture were classified by the author as pronation-eversion and 2 MFs without a PM fracture as supination-eversion injuries. However, he did not mention the displacement of fragments and described the results of treatment only in general terms. He recommended the following: “conservative treatment of patients with more advanced stages of the Maisonneuve fracture complex may be indicated, providing that the deltoid ligament and the medial malleolus are intact“.

Merrill et al. [6], in 1993, evaluated a series of 9 MFs with a mean follow-up of 26 months, without mentioning displacement of the fractures. They found a PM fracture in 4 patients and an MM fracture in 1 patient. In 8 patients, DL injury was suspected due to tenderness and swelling. Only 1 patient underwent surgery; the others were treated nonoperatively. An excellent result was recorded in 6 patients, good in 2 patients, and fair in 1 patient. The result for the operatively treated patient was not specified.

Neither author used CT or MRI for diagnosis [5, 6]. In the last 15 years there appeared 6 case reports dealing with nonoperative treatment of MF [15–19], which were based on MRI examinations and, in 2 cases, also on CT examinations (Table 2).

**Table 2:** Overview of published case reports

Autor	Year	NP	M/F	Age (y)	CT	MS	PM	ATFL	ITFL/ IOM	PTFL	FU (M)
Charopoulos	2010	1	1/0	68	-	-	+	+	nm	-	12
Van Wessem	2016	1	1/0	43	-	-	+	+	nm	+	17
Dietrich	2022	2	1/1	40/50	-	-/-	+/+	+/+	-/-	-/-	12
Jian Yu	2023	1	1/0	48	+	-	-	+	-	-	6
Wang	2024	1	0/1	20	+	DL	+	+	+	-	41

NP – number of patients, M/F – male/female, y -years, MS – injury of medial structures, DL – deltoid ligament, PM – fracture of the posterior malleolus, ATFL – injury of the anterior tibiofibular ligament, ITFL/IOM – injury of the interosseous tibiofibular ligament or the interosseous membrane, PTFL – injury of the posterior tibiofibular ligament, FU – follow up, M – months

Charopoulos et al. [15] described the case of a 68-year-old man who suffered an injury due to supination-external rotation trauma in mild plantar flexion of the foot. The patient was treated with a long-leg cast for 4 weeks and then with a short-leg cast for 2 weeks. One year after the injury, the patient had no complaints and stress radiographs showed no instability of the tibiofibular mortise.

Van Wessem et al. [19] reported three patients with MF, two of whom suffered a fracture of the fibula in the middle third. Only 1 patient, a 43-year-old man with a subcapital fracture of the fibula, met the criteria for MF. The duration of plaster cast immobilization was not specified. At a follow-up examination 17 months after the injury, the patient was completely free of symptoms.

Dietrich et al. [17] described 2 cases of a stable MF, a 40-year-old man and a 50-year-old woman, both of whom were treated with a short-leg walking cast for 6 weeks. At the follow-up one year after the injury, the AOFAS score was 90 and 82, respectively. The latter patient reported chronic ankle pain for several years, related to hyperlaxity dating from before the accident. Based on their experience, the authors defined the following criteria for nonoperative treatment of MF:

- Initial radiographs with an anatomical position of the mortise without diastasis,
- A fracture of the PM without talar subluxation or articular impaction,
- Persistence of an anatomic mortise position on follow-up weight-bearing radiographs,
- Absence of a secondary displacement of the PM on weight-bearing radiographs at follow-up,
- CT-scan or MRI showing a good position of the distal fibula in FN,
- Absence of DL rupture on MRI.

Jian Yu et al. [18] described 1 case of MF in a 48-year-old man. After short-leg cast therapy for 6 weeks, the AOFAS score reached 100 points at the 6-month follow-up after the injury.

Wang et al. [19] published a case of a 20-year-old woman with a MF assessed as unstable (rupture of the deltoid ligament and widening of the MCS) and indicated for surgery. The patient refused surgery and was treated with a short-leg cast for 8 weeks. In addition to MRI, a post-injury CT scan and two follow-up CT scans were performed. The axial post-injury scan showed widening of the TFCS, but without external rotation of the distal fibula. The first CT scan 3 months after the injury showed a decrease in TFCS values compared to the post-injury CT scan. The second CT scan, on the other hand, revealed an increase in TFCS compared to the first CT scan, but the “re-widening” did not reach post-injury values. Thirteen months after the injury, the AOFAS score reached 85 points, after 22 months 100 points, and after 41 months also 100 points. No signs of ankle osteoarthritis were found. The authors explained the excellent result of nonoperative treatment by the stabilizing effect of intact fibular (lateral) ligaments.

The results of nonoperative treatment in these case studies were rated as excellent in five cases; only one patient reported problems, which he had already experienced prior to the injury [17]. In our cohort, only 2 patients (N-8, N-13) had an AOFAS score of less than 90, and 11 patients achieved 100%. All patients had an FAOS score of more than 90, and 11 of them achieved 100%. Fracture of MM or PM was not associated with worse radiological and functional treatment outcomes in our cohort. Nevertheless, statistical analysis suggested that even partial injury to the syndesmosis and deltoid ligament, resulting in minimal displacement on CT scan, may worsen functional outcomes.

The nonoperative treatment method for most patients reported in the literature and all of our patients was a short-leg cast for 6 weeks, and none of the authors discussed the need for a longer period of fixation or reduced weight-bearing.

The mean age of our entire cohort was 61 years. This value is much higher than that of the literary cohort of 6 patients treated nonoperatively (45 years) [15–19] and the cohort of 100 MF patients (51 years) reported by Tuček et al. [14]. The difference is mainly due to the higher mean age of women (68 years) in our cohort.

Intact MS were found in 88% of our sample, and the results were similar in case reports (83%). However, in large MF samples, intact MS occurred in only 10% of cases [10].

PM fractures occurred in only 29% of our cases, whereas in the 6 case reports mentioned above, the incidence was 83% [15–19]. This corresponds to data from large studies, where the incidence of PM fractures ranged from 77 to 83% [9–11, 14].

In all 6 case reports, MRI always revealed a lesion of the anterior tibiofibular ligament (ATFL), of which 3 (50%) were avulsions of the T-Ch tubercle [17, 18]. We found intraligamentous lesions of the ATFL in all 6 patients examined by MRI, and diagnosed avulsion of the T-Ch tubercle on CT scans in 3 (18%) patients.

The position of the distal fibula in FN was mentioned in more detail only by Wang et al. [19]; other authors [15–18] have dealt with this problem only marginally. Assessment of the position of the distal fibula in FN is of fundamental importance in ankle fractures. However, this problem has not yet been reliably solved. Although there exist numerous methods, they are too complicated [24]. We used our own method developed on the basis of anatomical and clinical studies to evaluate the position of the fibula on axial CT scans taken 5 mm proximal to the joint line [25, 26].

Malposition of the distal fibula relative to the talus dome in MF described by Weber et al. [4] was not observed in our cohort; the anatomy of the lateral gutter was always normal. This can be explained by the stabilizing effect of all three fibular (collateral lateral) ligaments, which, when intact, maintain the normal relationship between the two bones.

For most physicians, the gold standard of treatment of MF is open reduction and fixation of the distal fibula in FN, including osteosynthesis of the associated fractures of MM and PM, as indicated. Subsequent early functional treatment or cast immobilization depends on the specific pathoanatomy of the fracture pattern [10, 14]. Published results are mostly very good, although complications such as malpositions or fractures of syndesmotic screws are more widely known [13].

MF stability is essential for choosing nonoperative treatment. According to our findings, this stability is characterized by intact MS or nondisplaced bicollicular fracture of MM, normal anatomy of the lateral gutter, and no or minimal malposition of distal fibula in FN. We consider these to be the limit criteria, as statistical analysis has shown a trend toward worse functional outcome in nonanatomical fibular malalignment and possible distension of the deltoid ligament.

In clinical practice, a stable MF should meet, in our view the following CT criteria: minimal malposition of the distal fibula in FN (widening up to 2 mm or external rotation up to 10 degrees); minimally displaced fracture of MM (up to 1 mm), or MCS less than 3 mm; minimally displaced fracture of PM (up to 2 mm). The criteria for nonoperative treatment have not yet been clearly defined; there is also a lack of comparison between conservative treatment with cast fixation and operative treatment with early rehabilitation.

We consider the strengths of our study to be the prospective follow-up of patients, complete CT documentation, a relatively large number of patients, and a longer follow-up period compared to previously published cases. Among the weaknesses of our study is the failure to perform stress/weightbearing radiographs or weightbearing CT to evaluate post-injury MCS and TFCS [27]. Last but not least, it would be appropriate to evaluate the outcome taking into account the pre-traumatic condition of the ankle. It cannot be ruled out that previous minor trauma, mild instability or discrete radiological changes, such as subfibular calcifications, may affect the final functional result, including ankle stability.

## Conclusion

The pathoanatomy of MF is highly variable. One variant is a stable fracture characterized by no or minimal displacement. However, this displacement must be assessed by CT scan. Our study has shown that the stable form of MF can be successfully treated nonoperatively.

## Data Availability

No datasets were generated or analysed during the current study.
